# Grain Boundaries Control Lithiation of Solid Solution Substrates in Lithium Metal Batteries

**DOI:** 10.1002/advs.202409275

**Published:** 2024-12-04

**Authors:** Leonardo Shoji Aota, Chanwon Jung, Siyuan Zhang, Ömer K. Büyükuslu, Aparna Saksena, Ezgi Hatipoglu, Poonam Yadav, Mahander Pratap Singh, Xinren Chen, Eric Woods, Christina Scheu, Se‐Ho Kim, Dierk Raabe, Baptiste Gault

**Affiliations:** ^1^ Max Planck Institute for Sustainable Materials 40237 Düsseldorf Germany; ^2^ Department of Materials Science and Engineering Pukyong National University 45 Yongso‐ro, Nam‐gu Busan 48513 Republic of Korea; ^3^ GTT‐Technologies 52134 Herzogenrath Germany; ^4^ Department of Materials Science and Engineering Korea University Seoul 02841 Republic of Korea; ^5^ Department of Materials Imperial College London London SW7 2AZ UK

**Keywords:** atom probe tomography, chemomechanical, grain boundary, lithiation, solid solution

## Abstract

The development of sustainable transportation and communication systems requires an increase in both energy density and capacity retention of Li‐batteries. Using substrates forming a solid solution with body‐centered cubic Li enhances the cycle stability of anode‐less batteries. However, it remains unclear how the substrate microstructure affects the lithiation behavior. Here, a correlative, near‐atomic scale probing approach is deployed through combined ion‐ and electron‐microscopy to examine the distribution of Li in Li‐Ag diffusion couples as model system mimicking high current densities. It is revealed that Li regions with over 93.8% at.% nucleate within Ag at random high‐angle grain boundaries, whereas grain interiors are not lithiated. The role of kinetics and mechanical constraint from the microstructure over equilibrium thermodynamics in dictating the lithiation process is evidenced. The findings suggest that grain size and grain boundary character are critical to enhance the electrochemical performance of interlayers/electrodes, particularly for improving lithiation kinetics and hence reducing dendrite formation.

## Graphical abstract

Grain boundary lithiation is responsible for the fast lithiation kinetics in solid solution substrates for lithium metal batteries. The preferential grain boundary lithiation creates a chemomechanical effect, localizing stresses near such interfaces and causing plastic deformation via a variety of mechanisms ranging from dislocation slip until grain boundary sliding.

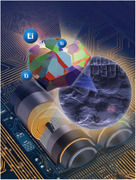

## 1 Introduction

With the increasing demand for energy storage from renewable sources,^[^ ^1^ ^]^ batteries have become vital to a transition toward a more sustainable society. Among many applications, lithium‐ion batteries stand out due to their high energy density, especially suitable for electric vehicles and electronic devices.^[^ ^2^, ^3^ ^]^ The increasing demand for Li‐ion batteries drives the improvement of their energy density, power, and capacity retention while employing cheaper and more sustainable materials.^[^ ^1^, ^2^ ^]^

Currently, graphite anodes are dominating the commercial market. Although graphite offers stable cyclability, it exhibits a limited gravimetric capacity of only 372 mAh g^−1^.^[^ ^4^ ^]^ Additionally, graphite shortage can cause supply disruptions in the near future,^[^ ^5^ ^]^ while synthetic graphite is neither scalable nor sustainable. The ideal anode material would be metallic Li, which exhibits the highest gravimetric capacity (3860 mAh g^−1^), and the lowest electrochemical potential of −3.04 V versus standard hydrogen electrode^[^ ^6^ ^]^ among all anodes. However, metallic Li suffers from poor cycle stability due to extensive side reactions with the electrolyte,^[^ ^7^ ^]^ as well as dendrite formation and growth causing short circuits and leading to battery failure.^[^ ^8^ ^]^

A promising alternative is to use lithiophilic metal substrates that reduce the metallic Li nucleation energy,^[^ ^9^, ^10^, ^11^, ^12^ ^]^ while providing high bulk and surface Li diffusivities.^[^ ^9^, ^10^, ^13^ ^]^ These substrates are reported to act as sinks for Li due to their high solid solubility,^[^ ^11^ ^]^ while homogenizing the Li distribution,^[^ ^14^ ^]^ thereby avoiding dendrite formation. Elements with high solid solubility in body‐centered cubic lithium (BCC‐Li), such as Mg, Al, and Ag^[^ ^12^ ^]^ could replace or simply coat current collectors.

The use of Ag‐based thin layers, usually as a composite with carbon, significantly improves the energy density and the cycle stability of anode‐less Li‐metal batteries, i.e., the Li anode is plated during lithiation.^[^ ^15^ ^]^ Despite Li plating/stripping being the main process underpinning the performance of these batteries, the lithium ingress into the substrate and Li removal from the substrate, respectively, occur simultaneously.^[^ ^11^ ^]^ In most materials, lattice defects control many materials’ properties, particularly elementary atomistic transport.^[^ ^16^, ^17^, ^18^ ^]^ Previous attempts to understand the effect of the substrate's microstructure, i.e., the hierarchy of material grains and defects, on Li plating/stripping^[^ ^19^ ^]^ and (de)alloying of the Li‐solid solution,^[^ ^20^ ^]^ focused on second phases formation. Insofar, grain boundaries had only been hypothesized to act as fast Li diffusion paths during lithiation.^[^ ^21^ ^]^ Previous studies could not directly resolve the spatial distribution of Li, and experimental results were interpreted based on equilibrium thermodynamics to understand phase transformations during (de)lithiation.^[^ ^10^, ^22^ ^]^

Here, we introduce a novel workflow that combines Li‐Ag micro‐diffusion couples made directly in situ in a scanning electron microscope–focused ion beam instrument (SEM‐FIB) with (scanning) transmission electron microscopy ((S)TEM) and atom probe tomography (APT) characterization. APT measurements provide 3D compositional maps with sub‐nanometer resolution^[^ ^23^, ^24^ ^]^ and the ability to discern up to 80% of all the ions a material consists of according to their mass‐to‐charge, also including light elements such as Li,^[^ ^23^, ^25^ ^]^ whereas crystallographic and structural information is provided by (S)TEM.^[^ ^26^, ^27^ ^]^ In addition, electron energy loss spectroscopy (EELS) was performed to visualize the Li distribution on a larger scale. Our Li/Ag model diffusion couples allow for the quantification of Li at grain boundaries to understand their role on lithiation, while avoiding secondary factors, such as the nature of the electrolyte, and environmental contamination.^[^ ^28^, ^29^, ^30^, ^31^ ^]^ Our results demonstrate that the lithiation is kinetically controlled by grain boundary diffusion, with confined formation of Li‐rich regions, containing up to >93.8 at.%, and does not follow bulk equilibrium thermodynamics. Lithiation and phase transformations in the grain interior are kinetically and possibly chemo‐mechanically hindered, implying that the earlier reported electrochemically measured high Li‐diffusivities in Ag must result from the intense grain boundary or surface lithiation mechanism observed in this work. Random high‐angle grain boundaries are favorable sites for the nucleation of Li‐rich regions, and hence controlling the grain size and grain boundary structure emerges as an important design criterion for next‐generation substrates for anode‐less batteries.

## 2 Results

### 2.1 Diffusion Couple

**Figure 1:**
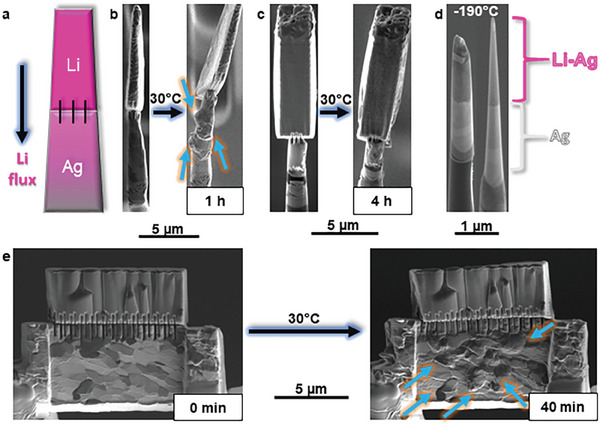
a) Ag micro‐pillar mounted on Si posts used for APT characterization. b) Micro diffusion couple of Li in contact with Ag at 30 °C. Three stripes are milled at cryogenic temperature (−190 °C) to ensure connection between both metals. After reacting for 1 h, the Ag pillar locally swells by 40–80% in volume near grain boundaries (blue arrows) due to the spontaneous lithiation. c) Micro diffusion couple with <100> Ag as substrate. After 4 h of reaction, no swelling but surface roughening is observed. d) Sharpening into APT specimens, with Li‐rich regions in dark contrast, and final APT specimen used for characterization. e) Diffusion couple on a TEM lamella of fine‐grained Ag, attached with 24 stripes to ensure connection. After 40 min, heterogeneous swelling is observed due to Li alloying.

A fine‐grained (average 1.5 µm) pure Ag (>99 at.%) and a <100> single‐crystal specimens taken from annealed (600 °C/5 h) Ag thin film were used as substrates for lithiation (Figure S1, Supporting Information). The detailed experimental procedure can be found in the Supporting Information. In short, Ag was lifted out and welded to a support in the FIB, and was then cut into micro‐pillar shaped specimens. Following surface cleaning, a Li‐lamella was attached on these substrates by using redeposition welding at −190 °C,^[^ ^32^ ^]^ forming micro‐diffusion couples at the contact, as illustrated in **Figure** **1**a. The stage was heated to 30 °C to trigger lithiation of the silver substrate. After 1 h, the fine‐grained Ag pillar non‐uniformly swells at certain grain boundaries (Figure 1b, right). The <100> Ag single crystal reacted with Li until 4 h without any volume expansion that could indicate alloying, Figure 1c. Finally, each Ag pillar is cooled down to −190 °C. APT specimens were finalized, as displayed in Figure 1d, so as to have their tip in selected sections of each pillar to monitor different stages of the reaction. A micro‐diffusion couple with fine‐grained Ag was made on a TEM lamella and subjected to lithiation for 40 min at 30 °C, leading to a volume expansion of 40–80%, Figure 1e, followed by cooling to −190 °C before thinning. Figures S2–S4 (Supporting Information) are snapshots of the lithiation process into specimens for APT and TEM.

### 2.2 Chemical Composition Evolution

**Figure 2:**
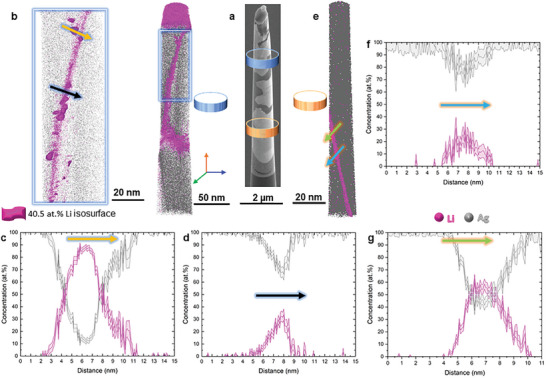
APT results of Li‐rich grain boundaries. a) Orange and blue rings indicate where APT measurements were performed in a typical micro‐diffusion couple. b) Top region, where three grains are visible. Some regions reach up to c) 87.6 at.% Li, while other areas along the grain boundary exhibit lower amount (down to 20 at.% Li), as indicated by the 1D concentration profile along the orange arrow. d) 1D chemical composition profile in another region, from the same grain boundary as (b), with more homogeneous Li distribution reaching 32.5 at.% Li in the black arrow from (b). e) A grain boundary ≈6 µm below the diffusion couple is visible from the high local Li concentration. 1D composition profiles in (f) through a precipitate‐free region of the same grain boundary, with up to 25.8 at.% Li (blue arrow in (e)) and g) through a Li‐rich precipitate containing up to 58.6 at.% Li (green arrow in (e)).

After 1 h of reaction at 30 °C, as shown in Figure 1 and Figures S2 and S3 (Supporting Information), the swelling of the Ag pillars is concentrated at the Ag grain boundaries, with local volume expansion typically ranging between 40% and 80% (Table S1, Supporting Information). The darker contrast in the electron micrograph in **Figure** **2**a indicates Li‐enriched regions at grain boundaries. At ≈2 µm below the diffusion couple interface, the APT analysis in Figure 2b evidences a heterogeneous Li distribution within a single grain boundary. Composition profiles through a precipitate, presented in Figure 2c, show that the Li content reaches values up to 87.6 at.% (orange arrow in Figure 2b), while the surrounding Li‐rich grain boundary in Figure 2d shows a decoration value near 32.5 at.% Li (black arrow in Figure 2b). In a zone 4 µm further below, another APT dataset also shows Li enrichment in a GB, Figure 2e. The Li content in this region is ≈25.8 at.%, Figure 2f, and Li‐rich precipitates contain near 58.6 at.% Li, Figure 2g. Regardless of the distance to the diffusion couple, the Li‐rich precipitates are nearly spherical in shape.

**Figure 3:**
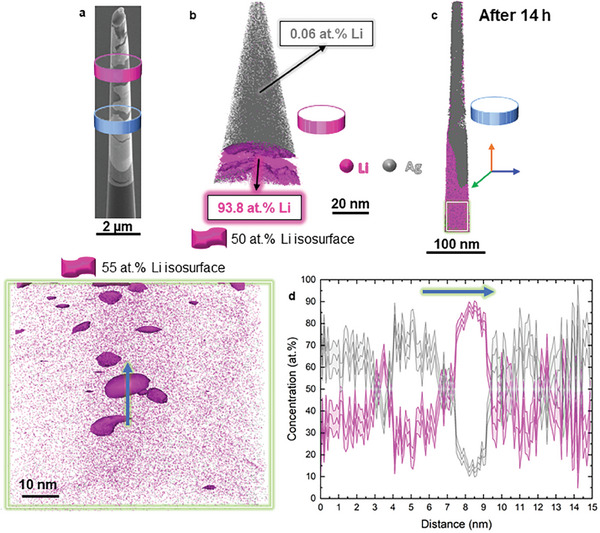
a) The approximate regions from where APT measurements were performed are shown by the blue and magenta rings in a typical micro‐diffusion couple. b) A second region exhibits a Li‐rich phase nucleated at a former Ag grain boundary reaching up to 93.8 at.% Li while the surrounding Ag grains remain non‐lithiated. c) APT data reconstructions from an interface between lithiated and non‐lithiated regions after 14 h reaction. The lithiated region is magnified in the green box (c), with spherical precipitates highlighted by the 55 at.% Li isosurface. d) A region of interest along one of the spherical precipitates shows the chemical composition reaching up to 88.2 at.% Li in a 1D chemical profile (blue arrow).

In other regions, such as shown in **Figure** **3**b, the Li content reaches values of even up to 93.8 at.%, indicating possible formation of a BCC‐Li nucleus on the grain boundary. The interface is sharp between the Li‐rich region and the adjacent FCC‐Ag bulk grain interior, which contains only below 0.06 at.%Li. After 14 h of reaction, Li‐rich regions formed along the grain boundaries imaged in Figure 3c contain 35–40 at.% Li, as quantified in the composition profile in Figure 3d. Nearly spherical precipitates are also distributed within these bulkier Li‐rich phases, and contain up to 88.2 at% Li, Figure 3d, while a remaining unreacted matrix still persists.

### 2.3 Microstructural Evolution

**Figure 4:**
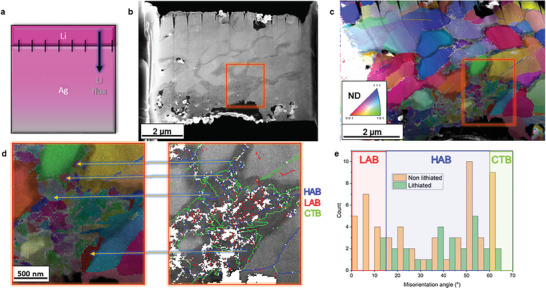
a) Schematic from the diffusion couple setup and the corresponding position of Li and Ag relative to the TEM/TKD lamella. The Li previously present at the top of the Ag was removed prior to the lamella thinning. b) Overview of the TEM lamella used for Transmission Kikuchi diffraction. Darker regions correspond to Li‐rich areas, which percolate along the grain boundaries. c) Inverse pole figure color scale (directions perpendicular to the observed surface, or ND – normal direction) with EDS Ag signal gray scale (darker regions indicate lower Ag amount, i.e., Li‐rich regions). d) Zoom‐in from the red box in Figure 4b,c displaying the TKD map left and the EDS Ag map (signal as gray scale) right with the corresponding grain boundary map overlapping. HAB: high angle boundaries in blue; LAB: low angle boundaries in red; CTB: coherent twin boundaries in green. e) Relationship between the misorientation angle and the observation of lithiation in a grain boundary. Low angle (ϴ ≤ 15°, red) and coherent twin grain boundaries (green) are mostly not lithiated, whereas high angle grain boundaries are mostly lithiated (ϴ > 15°).

In the scanning electron microscope, **Figure** **4**a,b, we used a combination of transmission Kikuchi diffraction (TKD) and X‐ray energy‐dispersive spectroscopy (EDS) on a diffusion couple created on the TEM lamella shown in Figure 1e. The darker regions in the backscattered electrons image, enriched in Li, percolate along the grain boundaries, leaving numerous grain interiors non‐lithiated even though they were close to the diffusion couple interface. There is also a highly lithiated region near the bottom of the sample. Figure 4c is the superimposition of the colored out‐of‐plane inverse pole figure map obtained through crystallographic analysis of the FCC‐Ag TKD patterns and an Ag EDS map in grayscale. In correlation, these maps reveal both, the structure and the chemical features with local resolution. Complementary selected‐area electron diffraction (SAED) lattice structure analysis conducted in the TEM, in Figure S5 (Supporting Information), supports the rationale that the lithiated region is mostly the FCC‐Ag phase, with a low volume fraction of Ag_3_Li precipitates usually seen agglomerated together (Figure S5b, Supporting Information). The lithiation process refines the grains (372 ± 202 nm) compared to the non‐lithiated areas (1465 ± 581 nm) as readily visible in the close‐up in Figure 4d. The correlation of the misorientation angle of the grain boundaries with the Ag EDS signal suggests that random high‐angle grain boundaries are preferentially lithiated; twin and low‐angle grain boundaries (which consist of dislocation arrays) show negligible tendency for lithiation (Figure 4e).

**Figure 5:**
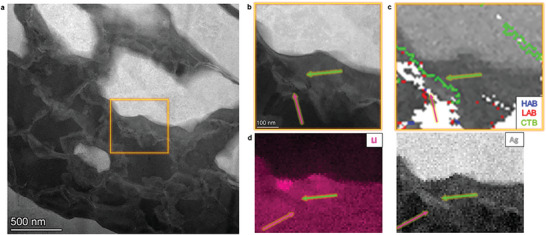
Ag‐Ag/Li interface observed by TEM, TKD, and EELS. a) Low magnification and b) zoom‐in HAADF images; c) Boundary map from TKD. HAB: high angle boundaries in blue; LAB: low angle boundaries in red; CTB: coherent twin boundaries in green. Li‐rich regions are visible as nanometric darker precipitates in (b). d) EELS maps from Li and Ag.

At higher resolution, high‐angle annular dark field (HAADF)‐STEM of the lithiated regions near the Ag/Li‐Ag interface in **Figure** **5**a shows Ag‐rich grains, with a bright contrast, and Li‐rich regions appear darker. Figure 5b is a close‐up on the region of interest marked by an orange box in Figure 5a, which contains dark nanometric features, with one indicated by the magenta arrow. The green arrow marks the region where a twin boundary is expected from the grain boundary map in Figure 5c. The contrast in the EELS map indicates a possible compositional shift in the twinned region (Figure 5d). The corresponding EELS spectrum for Li‐rich and Ag‐rich regions is shown in Figure S6 (Supporting Information). Small Li‐rich features are found near the Ag/Li‐Ag interface. Unfortunately, their crystal structure could not be resolved due to their small size and low volume fraction and would require higher resolution investigation at lower temperature to minimize damage from the electron beam. Similar Li‐rich features observed in the APT data (Figure 2c) reached up to over 88 at.% Li.

## 3 Discussion

### 3.1 Kinetics Versus Thermodynamics of Grain Boundary Lithiation

The Li enrichment at high‐angle grain boundaries can be predominantly caused by either thermodynamic or kinetic factors. Despite the observation of Li‐rich precipitates in the lithiated matrix, we consider only the FCC‐Ag phase for the thermodynamic calculations since the grain boundary lithiation in this phase acts as precursor mechanism for the precipitation. We assume that the adsorption isotherm leads to a near solid‐solution type of lithiation of the interfaces and, upon reaching a certain local chemical potential, interfacial precipitation can take place.

For the thermodynamic factor, equilibrium grain boundary segregation^[^ ^33^, ^34^, ^35^, ^36^ ^]^ could lead to a local Li‐enrichment due to energy minimization of the interface (hence, total) free energy through adsorption of solute atoms.^[^ ^37^ ^]^ This can be rationalized based on the enthalpy of mixing of the binary system, i.e., the excess enthalpy caused by the interaction between the segregating atoms in regular solutions,^[^ ^37^ ^]^ where a negative value indicates an exothermic reaction. The calculated enthalpy of mixing indicates a thermodynamic tendency to form a homogeneous solid solution (negative enthalpy) for FCC‐Ag across the entire Ag‐Li composition range (Figure S7a, Supporting Information), which is reflected in the wide FCC‐phase range in Figure S7b (Supporting Information).

This prediction is in contradiction with our experimental results in Figures 1, 2, 3, 4. Consequently, the observed preferential lithiation along grain boundaries cannot be elucidated through principles of bulk or respectively interfacial equilibrium thermodynamics in the solute limit alone. Rather, the quite extreme decoration (with a Li concentration of up to 93.8 at.% Li adjacent to unreacted Ag) and phase formation phenomena observed here seem to be dominated by kinetic (non‐equilibrium grain boundary segregation), i.e., enhanced grain boundary diffusivity, and possibly chemo‐mechanical factors. This is extremely likely to also be the case for lithiation with other electrode and electrolytes employed in batteries, especially when operated at higher current densities, which could further increase the Li flux and reinforce the importance of kinetics.

### 3.2 Role of Grain Boundary on Kinetics—Estimation of Li Diffusivity in Ag

**Figure 6:**
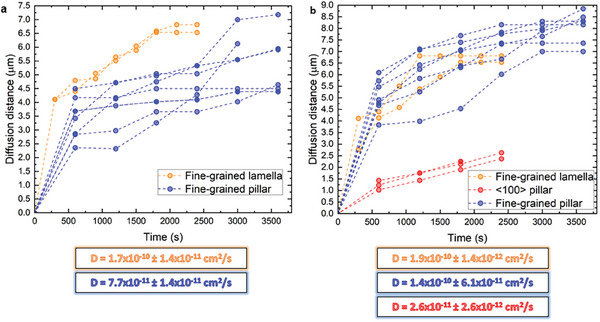
Li diffusion distance considering a) only Li alloying, i.e., the farthest swollen region observed. b) Li diffusion on the surface, where the propagation length was considered as the farthest region exhibiting some swelling or contrast change on the surface. <100> and fine‐grained pillars are considered, as well as fine‐grained lamella. Data from <100> single crystals are only available in (b) since they did not exhibit any swelling caused by Li alloying. The diffusion distance can become constant when it propagated along the whole length in (b) or if only a certain grain boundary was lithiated in (a). An example on the estimation of the diffusion distance is given in Figure S8 (Supporting Information).

The effect of grain boundaries on the Li diffusivity was quantified by the diffusion distance observed in the micro‐diffusion couple. Two scenarios are considered. First, we recorded the progression of the localized swelling associated to alloying, as plotted in **Figure** **6**a, ignoring the surface diffusion. Second, we considered contrast changes associated to the surface diffusion of Li. This was achieved by imaging a single location by using the ion beam as exemplified in Figure S8 (Supporting Information). The resulting evolution of the distance versus time is plotted in Figure 6b. Given the parabolic shape of the L versus t plot (L being the propagation length, t the time), the grain boundary/free surface lithiation of Ag is a diffusion‐controlled reaction.^[^ ^30^, ^31^, ^38^ ^]^ For such a reaction, the diffusivity can be calculated as the slope of L^2^ versus t plot or as L^2^/4t.^[^ ^38^ ^]^ We employed mainly the former scenario, except in the case where a single grain boundary was lithiated in Figure 6a, i.e., when the diffusion distance is constant after some time.

The <100> single crystal does not exhibit Li alloying even after exposure times of up to 4 h of reaction, as confirmed by APT measurements (Figure S9, Supporting Information); yet, Li still diffuses along the surface of the pillar, and we estimate a free‐surface diffusivity of D = 2.6 × 10^−11^ ± 2.6 × 10^−12^ cm^2^ s^−1^. We also find that Li diffusing through surface propagates farther than the alloyed regions, by a factor of ≈1.1–2 depending on the substrate's shape (Figure 6a,b). Pillars often contain only a single grain boundary that gets alloyed by the micro‐diffusion couple, and the constant diffusion distance in Figure 6a is due to the absence of a grain boundary network along which Li could diffuse farther, as exemplified in Figure 4.

Smaller cross sections, i.e., relatively larger surface/volume ratios, favor faster diffusivity due to the predominant effect of surface diffusion compared to the bulk.^[^ ^28^, ^30^, ^38^ ^]^ Yet the lamellar diffusion couple, Figure 1e, exhibits a longer diffusion distance, which can be rationalized by the presence of a grain boundary network, offering a fast diffusion path for Li. The similarity between diffusion coefficients for lamellas when comparing only alloying, D = 1.7 × 10^−10^ ± 1.4 × 10^−11^ cm^2^ s^−1^, with the surface diffusion of <100> pillar, D = 2.6 × 10^−11^ ± 2.6 × 10^−12^ cm^2^ s^−1^, is another evidence that diffusion through a grain boundary network can be as fast as free‐surface diffusion.^[^ ^39^ ^]^

### 3.3 Role of Lithiation‐Driven Grain‐Scale Mechanics—Kinematics, Plastic Deformation, and Strain Hardening

**Figure 7:**
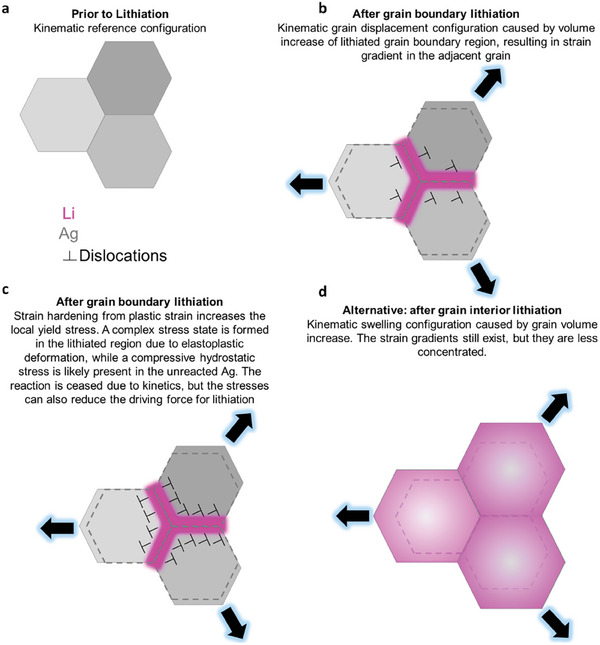
Schematics of the difference in grain kinematics depending on the lithiation mode. a) Reference configuration prior to lithiation. b) After grain boundary lithiation, a kinematic grain displacement takes place, caused by volume increase of lithiated grain boundaries, resulting in plastic strain in the adjacent unreacted grains. c) After a certain point of grain boundary lithiation, the plastic strain causes enough strain hardening to increase the yield stress of the adjacent Ag grains. This increases the hydrostatic stresses in the Ag grain and the triaxial stresses in the adjacent lithiated regions, reducing the driving force for further lithiation and ceasing the reaction. d) Alternatively, the kinematic swelling of grains would take place if the lithiation was taken mainly in the grain interior.

The increased Li content in the Ag anode as the lithiation proceeds has two major consequences on the microstructure. First, it creates a strain gradient between the Li‐rich and Li‐poor regions, as the crystalline FCC structure locally expands by 40–80 vol.% to accommodate the inserted Li. This massive volume increase in the chemically highly decorated regions along the grain boundaries causes a kinematic displacement perpendicular to the interface planes, as schematically depicted in **Figure** **7**a–c. Such kinematic displacement acting on the adjacent grains is different from the kinematic states observed when the whole bulk grain is lithiated (see the grain swelling in Figure 7d), which in turn creates a less intense local stress state in the adjacent, unreacted Ag grains.

**Figure 8:**
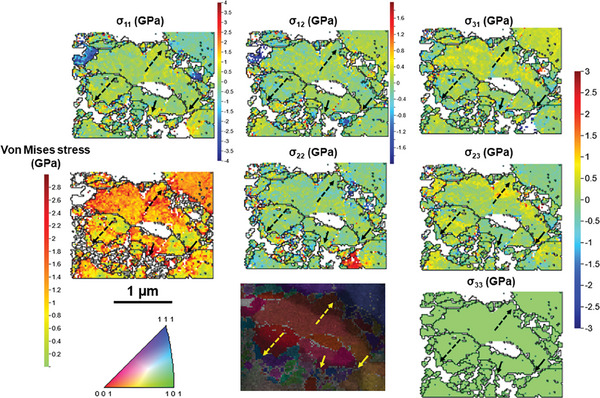
Local elastic stresses calculated from cross‐correlation, indicating higher von Mises stresses near the interface between lithiated and unreacted Ag grains, as indicated by arrows crossing such interfaces in all images. A complex stress state is caused by the volume expansion of the lithiated regions and the elastoplastic behavior of both lithiated and unreacted regions. Compressive stresses are negative, while positive stresses are tensile.

The strain gradient at lithiated grain boundaries is accommodated by arrays of geometrically necessary dislocations (GND). These defect patterns can be quantified in terms of the kernel average misorientation (KAM) values obtained from TKD. The KAM value is a metric for local orientation gradients that result from the volume mismatch, as displayed in **Figure** **8**a. The average GND density in the Ag grains adjacent to the Li‐rich areas at GBs is 7.6 × 10^14^ m^−2^ (see Supporting Information), comparable to severely deformed Ag.^[^ ^40^ ^]^ Extra statistically stored dislocations are also nucleated, although they do not contribute to the accommodation of strain gradients.^[^ ^41^, ^42^, ^43^ ^]^

The unreacted Ag grains prevail even after 14 h Li exposure (Figure 3c) and the high dislocation density in regions adjacent to the massively lithiated grain boundary areas indicates that a mechanical effect (elastic stresses) may aid in hindering the reaction within the grain interior. Elastic stresses are caused by misfit, such as differences in lattice parameter, heterogeneity in plastic deformation, differences in thermal contraction/expansion, or strain caused by phase transformation.^[^ ^44^ ^]^ After the grain boundaries are favorably lithiated due to the enhanced kinetics following a diffusion‐controlled reaction (Figure 6), the reaction switches to an interface‐controlled mode for the lithiation in the grain interior. This transition in the reaction mode occurs because of the strain hardening that results from the intense plastic activity in the adjacent Ag grain, as indicated by the high dislocation density (Figure 7c).

A complex triaxial stress state happens in both, the lithiated and unreacted regions, due to the elastoplastic behavior and inelastic stress relaxation by dislocation nucleation and slip (Figure 8). The von Mises stress is a criterion for plastic strain onset, which converts a triaxial stress state into a single stress value to be compared with the yield stress of Ag under uniaxial stress conditions observed in a standard tensile test. A local higher von Mises stress suggests likewise higher residual elastic stresses.^[^ ^45^ ^]^

The “flat” interfaces highlighted by dashed arrows in Figure 8 indicate an elastic stress peak within the unreacted Ag, near the reaction interface, with components of compressive normal stress and shear (triaxial state). In contrast, the interfaces near the grain corners, crossed by solid arrows, exhibit a triaxial stress concentration within the lithiated region. In both cases, the shear stresses are caused mostly by the elastoplastic behavior concentrated at the interface. At the corners, the elastic stresses are caused mainly by geometrically necessary dislocation arrays within the lithiated region, while the compressive and shear stresses within the unreacted Ag near the flat interfaces reduce the driving force for further lithiation.

The lithiated phase is softer than the adjacent Ag,^[^ ^46^ ^]^ undergoing intense plastic activity, as indicated by the superior GND density within the lithiated phase and at the reaction interface in Figure S10a (Supporting Information). The stresses caused by the expanded soft lithiated regions may be below the yield stress of the remaining hardened Ag grains and cannot be released by plastic deformation. As a result, the Ag grains are only in the purely elastic regime, resulting in the increased compressive stress in the adjacent lithiated region, thus mechanically decreasing the driving force for further lithiation.^[^ ^47^, ^48^ ^]^

Second, the lithiation gradually increases the homologous temperature, i.e., the ratio between the experimental temperature condition (30 °C) and the actual melting point for the specific local chemical composition, as seen in Figure S7b (Supporting Information). All diffusion barriers scale with the local (concentration‐dependent) melting point, such as the vacancy formation and diffusion energy. At increased homologous temperatures, the increased diffusivity and vacancy generation activates alternative deformation mechanisms, such as grain boundary sliding (creep), while also enabling microstructure restoration mechanisms to decrease the defect density, raised by plastic activity, i.e., dislocation recovery^[^ ^49^, ^50^ ^]^ and recrystallization.^[^ ^51^, ^52^ ^]^

After intense dislocation activity in the lithiated region, caused by lithiation at lower Li contents < 55 at.% Li (< 0.42 T_m_), concomitant grain boundary sliding and dynamic recovery and recrystallization take place for higher Li contents, particularly at Li > 80 at.%. When grains slide against each other and rotate, some additional mechanism is required to maintain the compatibility between these grains, such as diffusional‐^[^ ^53^ ^]^ or dislocation‐mediated^[^ ^54^ ^]^ deformation near grain boundaries. The grain boundary sliding is characterized by preserving the initial grain shape and creating grain boundaries with serrated or zig‐zag morphologies,^[^ ^55^ ^]^ as observed in many grain boundaries in Figure 4.

While recovery reduces the defect density mainly through dislocation annihilation and rearrangement, recrystallization is based on the nucleation and expansion of “defect‐free” nuclei mediated by a moving high‐angle boundary.^[^ ^56^ ^]^ In FCC materials with medium‐to‐low stacking fault energy, twin boundaries are formed during recrystallization.^[^ ^57^, ^58^ ^]^ As observed in Figure S10b (Supporting Information), some grains with low grain average misorientation are formed within lithiated regions and exhibit a twin relationship with one of the adjacent grains. Meanwhile, they are separated from other grains with a high average misorientation by high‐angle boundaries. These features are characteristic from the growth accident model for twin boundary formation,^[^ ^59^ ^]^ where a twin boundary is left behind a moving (high angle) grain boundary.^[^ ^60^ ^]^ The increased Li content reduces the stacking fault energy of the matrix lithiated region^[^ ^61^ ^]^ thereby facilitating the formation of such twin boundaries during recrystallization.^[^ ^62^ ^]^

The gradually increasing plastic strain and homologous temperature with the progressive Li alloying in the lithiated area triggers dynamic recovery and recrystallization.^[^ ^63^ ^]^ Such combination of grain boundary sliding and the microstructure restoration mechanisms has been reported in other metallic alloys at homologous temperatures > 0.5 T_m_.^[^ ^64^ ^]^ The dynamic nature is caused by the concomitant occurrence of recrystallization with the continuous plastic deformation, where the fresh “strain‐free” grains are deformed as soon as they are formed.^[^ ^65^ ^]^

### 3.4 Generalization in the Context of a Battery

Despite the advantage of tracking the Li ingress at specific lattice defects with no contamination or under the influence of secondary factors, the use of diffusion couples has limitations when compared to a battery in operation. First, no electrolyte is directly used and any effect from the solid electrolyte interphase and electrolytes are not reproduced in our experiment, despite their strong influence on the electrochemical behavior.^[^ ^66^ ^]^

Second, there is no reduction of Li^+^ ions in a diffusion couple, as opposed to at an anode in an electrochemical battery system.^[^ ^67^ ^]^ In this case, the lithiation caused by the direct contact of metallic Li and Ag is purely driven by the difference in Li chemical potential and it is regulated only by Li^0^ diffusion in Ag. A diffusion couple represents the lithiation of the substrate once the current density in a battery is sufficiently high to form a continuous metallic Li layer on top of the Ag substrate. Applying an external potential during lithiation lowers the potential toward more negative values (or increases the chemical potential of Li), driving the formation of Li‐Ag phases with increased Li content depending on the applied current density.^[^ ^11^, ^22^, ^68^ ^]^ At some point, the potential would be negative versus Li^+^/Li and metallic Li would be plated in the Ag, as commonly observed in Ag^[^ ^10^, ^11^ ^]^ and Ag‐C^[^ ^15^, ^69^ ^]^ interlayers/substrates.

In the diffusion couples, we only observed FCC‐Ag and some minor Ag_3_Li phases (Figure S5, Supporting Information). The nucleation of Li‐rich particles or phases apparently follows this reaction sequence: FCC Ag‐Li → FCC Ag‐ (20‐40 at.%)Li → likely BCC phase with up to 93.8 at.% Li. The transformation sequence matches with the predicted stable phases at room temperature in a revisited Ag‐Li phase diagram.^[^ ^70^ ^]^ Our experimental results indicate that over‐saturation^[^ ^71^ ^]^ of the FCC phase does not take place. Instead, the spherical shape of Li‐rich precipitates within the lithiated boundaries indicates that BCC phase should nucleate by a classical two‐phase mechanism. Despite the strong kinetic and mechanical effect during the overall lithiation, the local phase transformation pathway within a single grain boundary appears to follow a near‐equilibrium condition since it is kinetically allowed.

The diffusion at grain boundaries should follow the A‐C classification from Mishin,^[^ ^72^ ^]^ where in the C regime the diffusion is constrained to the grain boundary plane. After prolonged times, the Li diffuses toward the grain interior, reaching the adjacent regions from the grain boundary (B) and, finally, consuming the whole grain (A). This sequence causes the kinematic grain displacement (Figure 7b,c), resulting in confined local plastic strain at the Ag grains adjacent to the lithiated boundaries, possibly aiding at ceasing the reaction in regime C after a certain elastic stress is achieved. With an applied potential, we expect that grain boundaries will still be preferentially lithiated at first given kinetic and likely chemomechanical reasons, a mechanism that intensifies at higher current densities.

As observed in Figure 4e, not all grain boundaries are effective to host Li and later nucleate Li‐rich regions. Only high‐angle grain boundaries (ϴ ≥ 15°) are effectively preferentially lithiated due to their higher energy and higher diffusivity. Low angle boundaries (2 < ϴ < 15°) and coherent twin boundaries, characterized by lower boundary energy^[^ ^73^ ^]^ and lower diffusivity,^[^ ^74^ ^]^ are not lithiated. This is detrimental for lithiation and Li transport due to two factors. First, a lower boundary energy is detrimental for the ability of a grain boundary to reduce the nucleation overpotential^[^ ^75^ ^]^ by heterogeneous nucleation. Second, the lower grain boundary energy causes a lower tendency for grain boundary enrichment,^[^ ^76^ ^]^ and hence lower grain boundary diffusivity of Li, hindering the Li transport within the substrate.

Despite the lower electrical conductivity of grain boundaries,^[^ ^77^, ^78^, ^79^, ^80^ ^]^ high‐angle grain boundaries/electrolyte interfaces also act as preferential nucleation sites for Li^+^ reduction and alloying by being more effective in reducing the effective surface energy. Such behavior is confirmed when electrochemically lithiating Ag thin films in liquid electrolyte, as demonstrated in Figure S11 (Supporting Information). At higher current densities, Li plating may gradually instensify on the bulk grain/electrolyte interfaces due to the lower electrical conductivity of grain boundaries. Yet, the predominant effect of enhanced Li diffusivity at random high‐angle grain boundaries, characterized by a more disordered structure, would be beneficial with both liquid and solid electrolytes. In liquid electrolytes, it would attract Li from the metallic Li layer into the substrate, while it would offer fast Li diffusion paths for the Li deposition between the substrate and the current collector in solid electrolytes.^[^ ^70^ ^]^ As such, increasing the random high‐angle grain boundary density could accelerate the overall Li intake and offer more nucleation sites for homogeneous metallic Li plating and alloying, hence decreasing the propensity for dendrite growth and localized alloying.

An additional way of improving the electrode design to inhibit dendrite growth is to use active material as nano‐ or microparticles, instead of Ag is often used in the form of nanoparticles or in thin film/foils form. As seen in Figure S12 (Supporting Information), even the pillars with Ø < 1 µm exhibit a more intense lithiation due to lower mechanical constraints when compared to pillars with Ø > 1 µm. A composite electrode offers free volume for Ag nanoparticle expansion upon lithiation. In contrast, thin films or foils exhibit no free volume that could compensate for the gradual concentration‐dependent expansion of the Ag crystals. This would result in even enhanced and rather intense strain hardening and, consequently, high elastic stresses, associated also with associated plastic relaxation processes, i.e., nucleation and accumulation of dislocations, once the elastic limit is reached, a scenario likely to happen already at low loads. The lithiation of grain interiors would be more difficult and crack formation could take place to reduce these stresses, possibly also aided by local stress peaks that may build up caused by the presence of arrays of geometrically necessary dislocations as described above. Alternatively, a limited lithiation at grain boundaries could take place, possibly compromising the performance of thin film/foil.

If the electrochemical cycling is performed at a higher temperature (for example, up to 80 °C), the enhanced vacancy formation and Li diffusivity may allow crystalline defect annihilation. The resulting stress release could facilitate the lithiation of the grain interior, while the improved overall Li mobility would decrease the kinetic advantage of grain boundaries, tending toward a more homogeneous Li distribution.

Given the preferential grain boundary lithiation, this is also the region where delithiation should primarily be occurring. Most of the grain boundaries are observed in the lithiated region in Figure 4: those previously existing and those formed during lithiation. Two aspects are worth considering in this context. Thermodynamically, grain boundaries contain energetically favorable binding sites for Li.^[^ ^81^ ^]^ Upon delithiation, some of the Li therefore remains trapped inside these interfaces, resulting in capacity loss already during the 1st cycle. Kinetically, grain boundary diffusivity is generally faster than in the bulk, and in the abutting regions, the higher Li concentration and associated corresponding change in the local homologous temperature will likely also accelerate diffusion. The reversible capacity will be improved at higher temperatures, as thermal energy can release some of the trapped Li, in addition to further enhancing Li diffusivity.

The preferential grain boundary lithiation and the role of elastic stresses in inhibiting further lithiation of the grain interior highlight the need for further investigations. For example, improving the composite electrode mesostructure, including the microstructure of the active material, to reduce such elastic stresses. In addition, we will explore the potential for alloying Ag with solute which can segregate to the grain boundary, which can be predicted from thermodynamics. These solutes can fill the energy traps at grain boundaries prior to the first lithiation, thereby avoiding irreversible Li loss and improving the reversibility of the reaction. These thermodynamically stabilized grain boundaries would still offer the kinetic advantage for Li‐diffusion.

In summary, we used micro‐scale Li‐Ag diffusion couples to elucidate the critical role of grain boundaries in the local enrichment of Li on Ag substrates. Preferential grain boundary lithiation can also be expected in other substrates soluble in BCC‐Li, such as Mg and Al, as both undergo Li grain boundary segregation.^[^ ^82^, ^83^ ^]^ Our findings reveal that FCC Ag‐Li solid solutions predominantly form at random high‐angle grain boundaries (ϴ ≥ 15°), with Li concentration up to 40 at.%. Such Li‐enriched boundaries host spherical precipitates reaching up to ≈88.2 at.% Li, while some FCC‐Ag grain boundaries exhibit confined phases reaching >93.8 at% Li region. Low angle boundaries (2° < ϴ < 15°) and coherent twin boundaries are not lithiated due to their lower segregation energy and Li diffusivity. While Li did not show any alloying within a <100> Ag single crystal, the combination of grain boundary and surface diffusions yielded a superior diffusivity compared to surface diffusion alone. Thus, grain boundary character and density could be used to design optimized substrates/anodes for enhanced lithiation kinetics and decreased dendrite growth propensity.

The lithiation takes place mainly along the grain boundaries, without lithiation in most of the grain interiors. This effect leads to a corresponding kinematic grain displacement, indicating the predominance of a kinematic (position shifting) effect that translates to corresponding kinetics of dislocation populations which in turn mechanically reduces the driving force upon lithiation. It is unlikely that equilibrium thermodynamics alone can be used to predict the chemical evolution for any electrode/solid electrolyte material employed in a battery. Thus, to fully account the lithiation behavior, thermodynamics, mechanics and kinetics should be considered.

## 4 Experimental Section

### Diffusion Couple

A fine‐grained (average 1.5 µm) pure Ag (> 99 at.%) and an annealed (600 °C/5 h) Ag thin film were used as anode for the lithiation. Ag thin films were deposited on the surface of Si wafers, which had a 1000 nm thick wet oxide layer of SiO_2_. Films were deposited by magnetron sputtering from a high‐purity Ag target (99.99%) in a radio frequency (RF) gun within a physical vapor deposition (PVD) system (BESTEC, Berlin, Germany). The deposition was conducted at room temperature, achieving a film thickness of ≈1000 nm. The deposition rate was maintained at 0.6 nm s^−1^. Before the sputtering, the chamber was evacuated to a base pressure of 1 × 10^−6^ Pa. Argon gas was then introduced as the sputtering medium at a flow rate of 40 standard cubic centimeters per minute (SCCM), establishing a working pressure of 0.3 Pa. From the annealed Ag thin film, a 20 µm <100> grain was lifted out to produce <100> single‐crystal specimens (Figure S1, Supporting Information).

A Xe‐plasma focused ion beam (Thermo‐Fisher Helios PFIB) was used to lift out and mount the Ag specimen onto a series of commercial Si coupons, following the first steps of the protocol outlined in Ref. [84] as shown in Figure 1a. This coupon was transferred to a nitrogen glovebox, where a Li piece was cut and added onto a second holder. Both were transferred through a high vacuum suitcase (<10^−6^ mbar),^[^ ^85^ ^]^ at room temperature, into a ThermoFisher Helios 5 Ga‐FIB.

A Li‐lamella was lifted out under cryogenic conditions, using the protocol detailed in Ref. [32] After cleaning the surface of the top of the Ag pillar and the bottom of the Li lamella at 30 kV/0.23 nA, both metals were contacted, and subsequently four stripes (1.5 µm length) were milled (30 kV/24 pA) vertically at the Li/Ag interface to guarantee proper connection between the metals, Figure 1b, forming micro‐diffusion couples. A batch of six was made in one session. The stage was heated to room temperature and the silver lithiation took place after ≈30 min. After 1 h, the fine‐grained Ag pillar pronouncedly swelled and the Aquilos 2 (Thermo‐Fisher) stage was cooled down to −190 °C (Figure 1b, right), while the <100> Ag single crystal was reacted until 4 h (Figure 1c). The cooling takes ≈15 min. Each Ag pillar was sharpened into needle‐like specimen suitable for APT, as displayed in Figure 1d.

A similar micro‐diffusion couple was created on a lamella for TEM. An Ag lamella with dimensions 10 × 3 × 6 µm^3^ was mounted onto a Mo grid in the PFIB. The lamella was air transferred to the Ga‐FIB. Under cryogenic conditions in the Ga‐FIB, Li was lifted out and connected to the Ag lamella after surface cleaning. ≈24 stripes were vertically milled (1.5 µm length) to ensure proper contact and adhesion of the Li/Ag interface, Figure 1d, left. The stage was heated to 30 °C to activate the reaction and maintained at this temperature for 40 min. The Li alloying was visible from the swelling of the Ag lamella (Figure 1e, right). This was followed by cooling to cryogenic temperatures (−190 °C) for thinning, which takes ≈30 min. Figures S2–S4 (Supporting Information) were series of snapshots of the diffusion process into APT specimens and the TEM lamella, respectively.

The Li diffusion distance was estimated as the farthest distance from the Li/Ag interface where a microstructure change was observed in SEM. Two cases were considered: first, the case where Li alloying, indicated by swelling, and Li surface diffusion, characterized by contrast changes on the surface of the Ag substrate were accounted. In the second case, only alloying (farthest swollen region) was considered.

The local volume change was calculated considering a cylinder with constant height, where the swelling takes place mainly along the diameter. The diameter from a swollen region was measured before and after reaction and the initial and final volumes of the swollen regions were calculated as

(1) $$V=\pi r^{2}h$$

where *r* was the radius of the swollen region and h the height of the swollen region. From both initial and final volumes, the volume expansion (∆V) was calculated as

(2) $$\Delta V\left(\%\right)=\left(\frac{V_{f}-V_{0}}{V_{0}}\right)\times 100$$

where *V_f_* and *V_0_* were the final and initial volumes, respectively.

Some inaccuracies can arise from the rotation and bending of the tip caused by the swelling. This can cause the comparison of before/after diameter in slightly different locations. The height was not precisely constant after lithiation, but it cannot be precisely measured due to the aforementioned rotation/bending of the pillar. The obtained values usually range between 40% and 80% in volume, although two samples exhibit pronounced local swelling, reaching up to 408.8 vol.% in Table S1 (Supporting Information). This was likely caused by a lower mechanical constraint in this pillar, the only one with an initial diameter below ≈1 µm, as indicated by the heavy bending when compared to the other pillars shown in Figure S12 (Supporting Information). The lower mechanical constraint allows for enhanced local lithiation at the grain boundaries and also at the adjacent grains. In addition, since a uniform swelling through the pillar's cross‐section was considered, the quantified 408.8 vol% expansion was likely slightly overestimated due to the localized swelling on one side of the pillar.

### Ag Thin Film and Electrochemical Lithiation

Ag thin films were synthesized using direct current magnetron sputtering at room temperature (without intentional heating). The chamber schematics of which can be found in.^[^ ^86^ ^]^ The experiment was performed in argon atmosphere of 0.4 Pa while the base pressure, before the deposition, was maintained ≤7 × 10^−5^ Pa. The sample holder was rotated to obtain homogenous thickness over 304 stainless steel substrates. A target power density of 2.5 W cm^−2^ was applied on the Ag target for 45 min to reach the desired thickness of 1.5 µm. These 10 × 10 cm^2^ thin films were used as anode. 150 µL of 1 m LiPF_6_ in EC:DMC (ethyl carbonate:dimethyl carbonate) was used in a 2032 coin cell, with a Ø = 12 mm Li counter electrode and a Al_2_O_3_‐coated PP (polypropylene) separator. Galvanostatic lithiation was performed at 50 µA cm^−2^ until 0.58 mAh cm^−2^ capacity was achieved. The sample was transferred and cross‐sectioned similar to all the other samples inside the Ga‐FIB.

### Atom Probe Tomography

Atom probe tomography measurements were performed in a local electrode atom probe LEAP 5000XS (Cameca Instrument Inc.) using the pulsing laser mode. The laser energy was 50–60 pJ, at a pulse repetition rate of 200 kHz with a target detection rate of 1 ion per 200 pulses on average, at a base temperature of 50 K. Data reconstruction and processing was performed in CAMECA's APSuite 6.3.

### Transmission Electron Microscopy

Transmission electron microscopy and selected area electron diffraction were acquired with an image‐corrected Titan Themis microscope (Thermo Fisher Scientific) operated at an acceleration voltage of 300 kV. Scanning transmission electron microscopy (STEM) was performed at 300 kV on a probe‐corrected Titan Themis microscope (Thermo Fisher Scientific). For high‐angle annular dark field (HAADF)‐STEM imaging, inner‐outer collection angles of 73–200 mrad were used. Electron energy loss spectroscopy (EELS) spectrum imaging was acquired using a Quantum ERS spectrometer (Gatan), and processed by multivariate statistical analysis.^[^ ^87^ ^]^

### Transmission Kikuchi Diffraction

Transmission Kikuchi diffraction (TKD) was performed in a Zeiss Merlin field emission SEM. The electron beam acceleration voltage and current were 30 kV and 2 nA, respectively. A 7.3 × 5 µm2 map was acquired, with a step size of 15 nm, with simultaneous X‐ray energy‐dispersive spectroscopy (EDS) measurement. The acquired data was treated in the EDAX OIM Analysis 8.6 software. Grain confidence index standardization was used as cleanup procedure, followed by removal of any measured pixel with a confidence index lower than 0.1.

The Ag EDS signal count exhibits a bimodal curve that was fitted with two Gaussian curves, and their intercept was used to separate the Li‐rich from the unreacted Ag grains. These regions were extracted to calculate the kernel average misorientation (KAM) and geometrically necessary dislocation (GND) density. The KAM increases linearly with the number of nearest neighbors considered in the kernel. The GND density was calculated based on the KAM. Since the GND density for a single sample should not vary depending on the user‐chosen parameter, the ϴ_true_ was calculated based on:^[^ ^88^, ^89^ ^]^

(3) $$\theta_{true}=\theta_{noise}+n\theta_{true}$$

where n was the size of the kernel (*n* = 1–4) and ϴ_noise_ was the intercept in the y‐axis of the linear fit. The average GND density was then calculated as:^[^ ^89^ ^]^

(4) $$\rho_{GND}=\frac{\theta_{true}}{b.n.a}$$

where b was the Burgers vector (b = 0.2892 nm for Ag), n was the size of the kernel (*n* = 3) and a was the measurement step size (a = 15 nm).

For cross‐correlation, a similar setup was used. A step size of 25 nm was used for a map of 9 × 5.5 µm2 with simultaneous EDS measurement. For each pixel, the Kikuchi diffraction pattern was recorded as an image. For cross‐correlation, the software CrossCourt 4 was used. Only points with CI > 0.1 were employed for the analysis, while only FCC‐Ag was detected. The elastic constants for both unreacted and lithiated Ag grains were considered the same, with C_11_ = 13.5 GPa, C_12_ = 11.44 GPa, and C_44_ = 8.78 GPa.

### Thermodynamic Calculations

The software FactSage 7.3 was used to calculate the mixing enthalpy as function of Li amount in the FCC phase from the Ag‐Li system with the FTLite database. The equilibrium phase diagram was also calculated for this system. The calculated negative enthalpy of mixing for the major FCC phase observed in the lithiated region, plotted in Figure S7b (Supporting Information), indicates a thermodynamic tendency to form a homogeneous FCC Ag‐Li solid solution across the entire Ag–Li composition range, i.e., without segregation. This was reflected in the large solid solution range for this phase in the calculated phase diagram, Figure S6c (Supporting Information).

## Conflict of Interest

The authors declare no conflict of interest.

## Supporting information



Supporting Information

## Data Availability

The data that support the findings of this study are available from the corresponding author upon reasonable request.
