# Supplementary material for: Grain Boundaries Control Lithiation of Solid Solution Substrates in Lithium Metal Batteries
Source: Adv Sci (Weinh). 2024 Dec 4;12(4):2409275. doi: 10.1002/advs.202409275 (PMC11789589; doi:10.1002/advs.202409275)
Supplement: Supplementary file 1 — Supporting Information [file ADVS-12-2409275-s001.docx]

**Grain boundaries control lithiation of solid solution substrates in lithium metal batteries**

Leonardo Shoji Aota^a,*^, Chanwon Jung^a,b^, Siyuan Zhang^a^, Ömer K. Büyükuslu^c^ , Aparna Saksena^a^, Ezgi Hatipoglu^a^, Poonam Yadav^a^, Mahander Pratap Singh^a^, Xinren Chen^a^, Eric Woods^a^, Christina Scheu^a^, Se-Ho Kim^a,d^, Dierk Raabe^a^, Baptiste Gault^a,e^

^a^ Max Planck Institute for Sustainable Materials, 40237, Düsseldorf, Germany

^b^ Department of Materials Science and Engineering, Pukyong National University, 45 Yongso-ro, Nam-gu, Busan 48513, Republic of Korea

^c^ GTT-Technologies, 52134, Herzogenrath, Germany

^d^ Department of Materials Science and Engineering, Korea University, Seoul 02841, Republic of Korea

^e^ Department of Materials, Imperial College London, London, SW7 2AZ, UK

* Corresponding author

**Supplementary Figures**

**
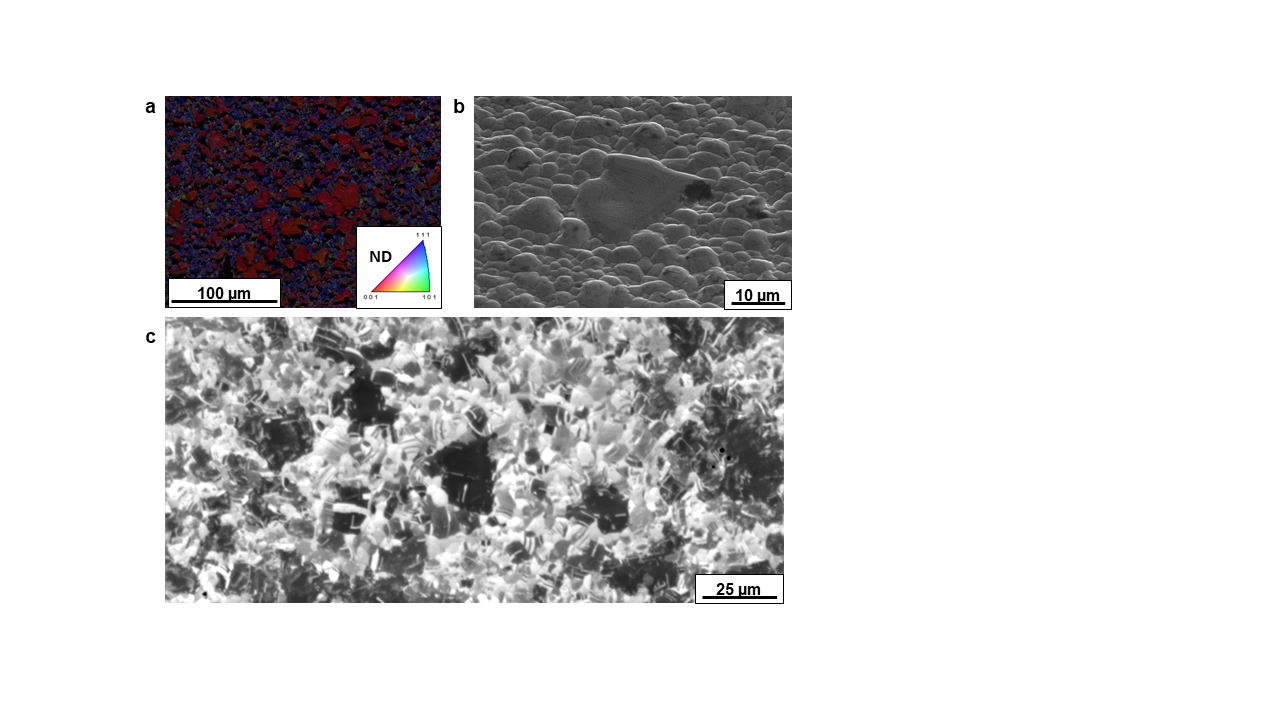
**

**Supplementary Figure S1 –** Ag thin film following annealing at 600°C/5 h. (a) EBSD map showing the coarser grains as <100> || ND (normal direction). (b) <100> grains are seen as protuberance at the surface, while the finer grains exhibit lower height. (c) In the Xe-ion beam (secondary electrons detector) image from the FIB-SEM, <100> grains appear as dark. The central grain was used as lift-out for <100> single crystals.

**
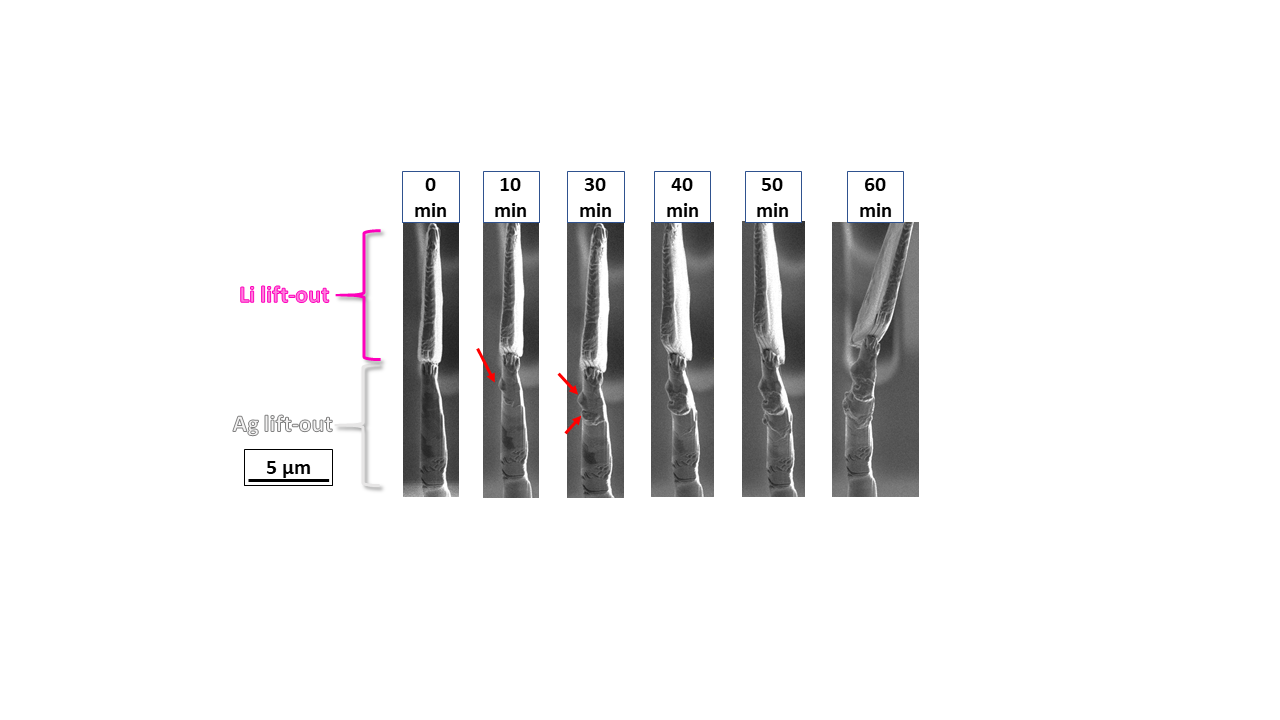
**

**Supplementary Figure S2 –** Li-Ag micro diffusion couple setup employing fine-grained Ag to produce atom probe tips inside the FIB. The magenta arrows highlight the swelling in the Ag substrate due to Li ingress. Some specific regions are intensively swollen, while others remain unreacted.


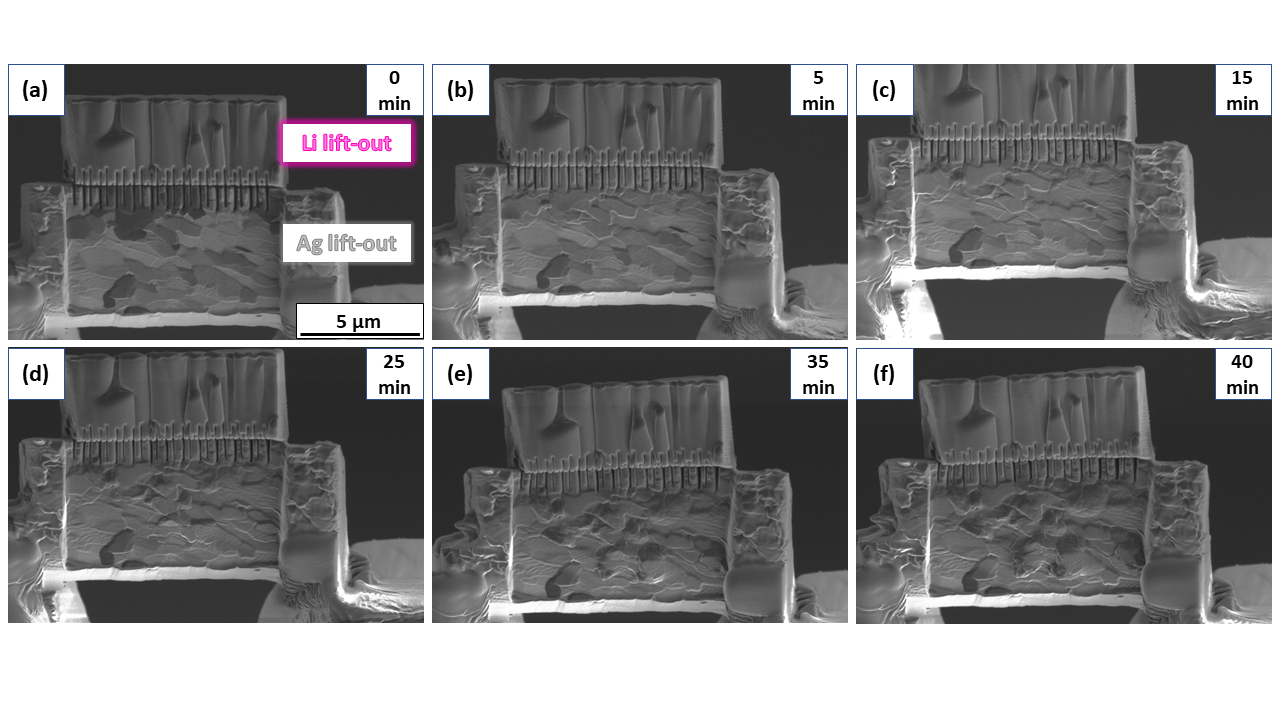


**Supplementary Figure S3 –** Ag-Li micro diffusion couple setup inside the FIB for a TEM lamella. Snapshots taken after a reaction time of (a) 0 min, (b) 5 min, (c) 15 min, (d) 25 min, (e) 35 min and (f) 40 min. Note the swelling of many specific grain boundaries and some grains.


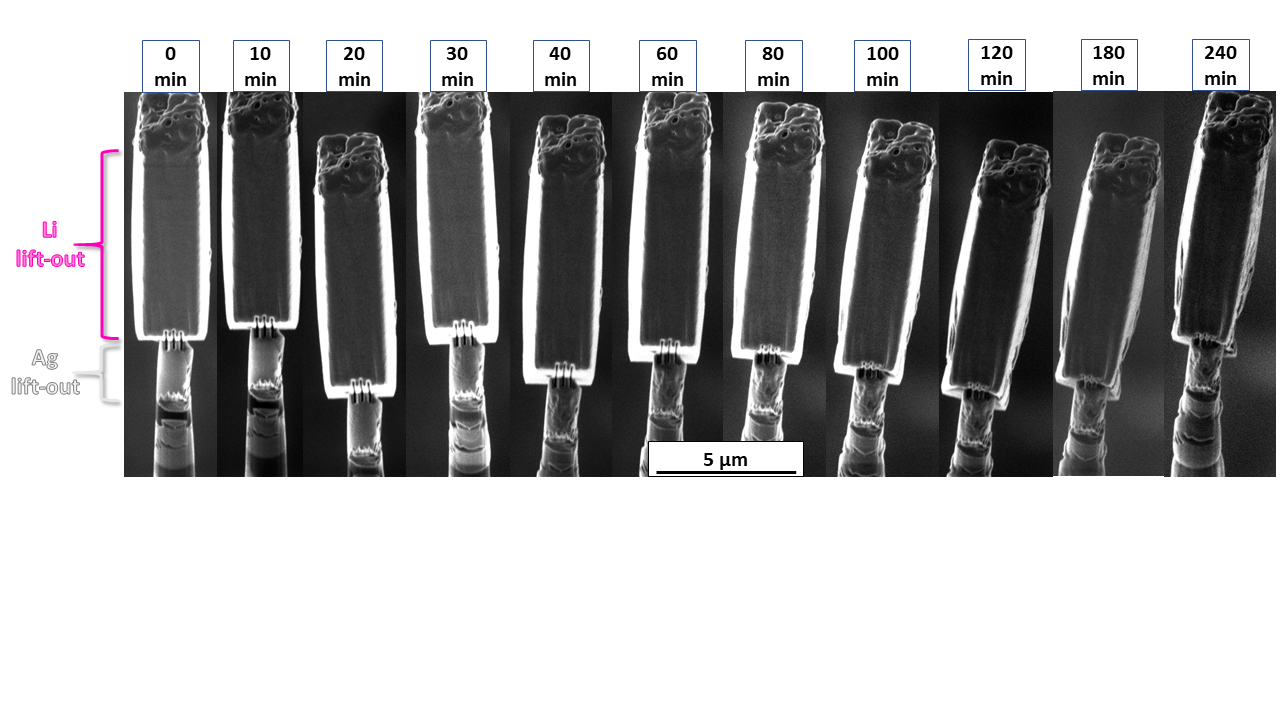


**Supplementary Figure S4 –** Ag-Li micro diffusion couple to produce APT tips from <100> Ag single crystal. The Ag single crystal does not swell, but only exhibits surface roughening, indicating Li diffusion and plating at the surface of the pillar without alloying.


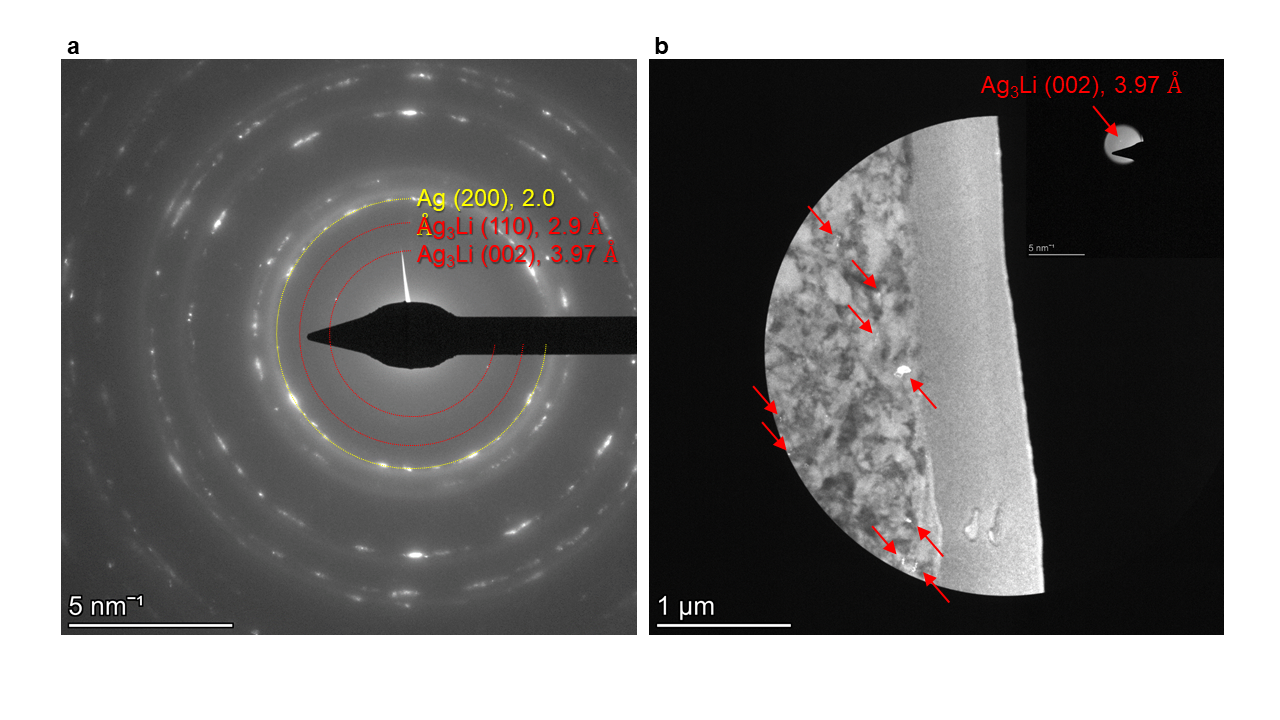


**Supplementary Figure S5 –** (a) Selected area electron diffraction pattern from the lithiated region. (b) Bright spots correspond to the isolated diffraction spot from Ag_3_Li phase. Note that this phase appears as clusters, whereas most of the lithiated region is composed of FCC-Ag.


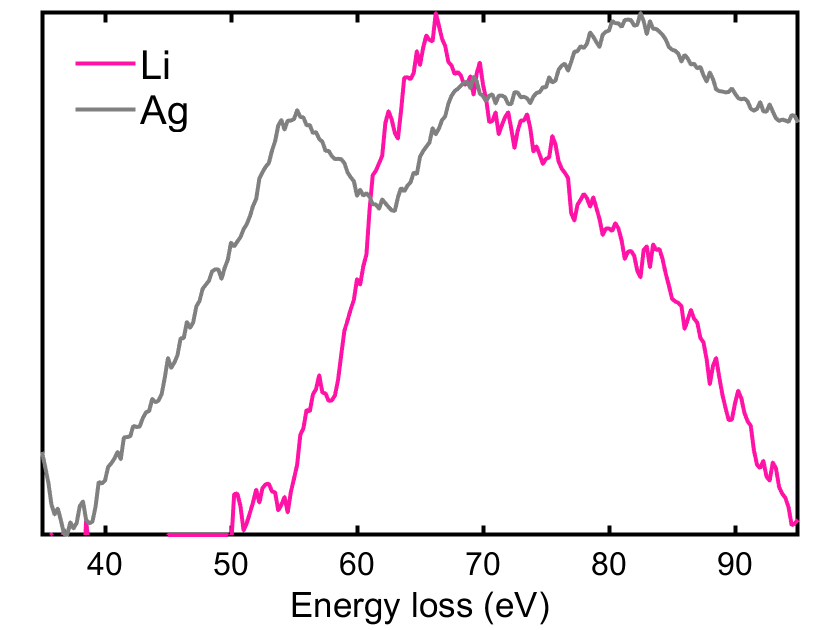


**Supplementary Figure S6 –** The electron energy loss spectrum for Li-rich and Ag-rich regions showing the Li-K and Ag-O2,3 edge from the area depicted in Figure 5d.


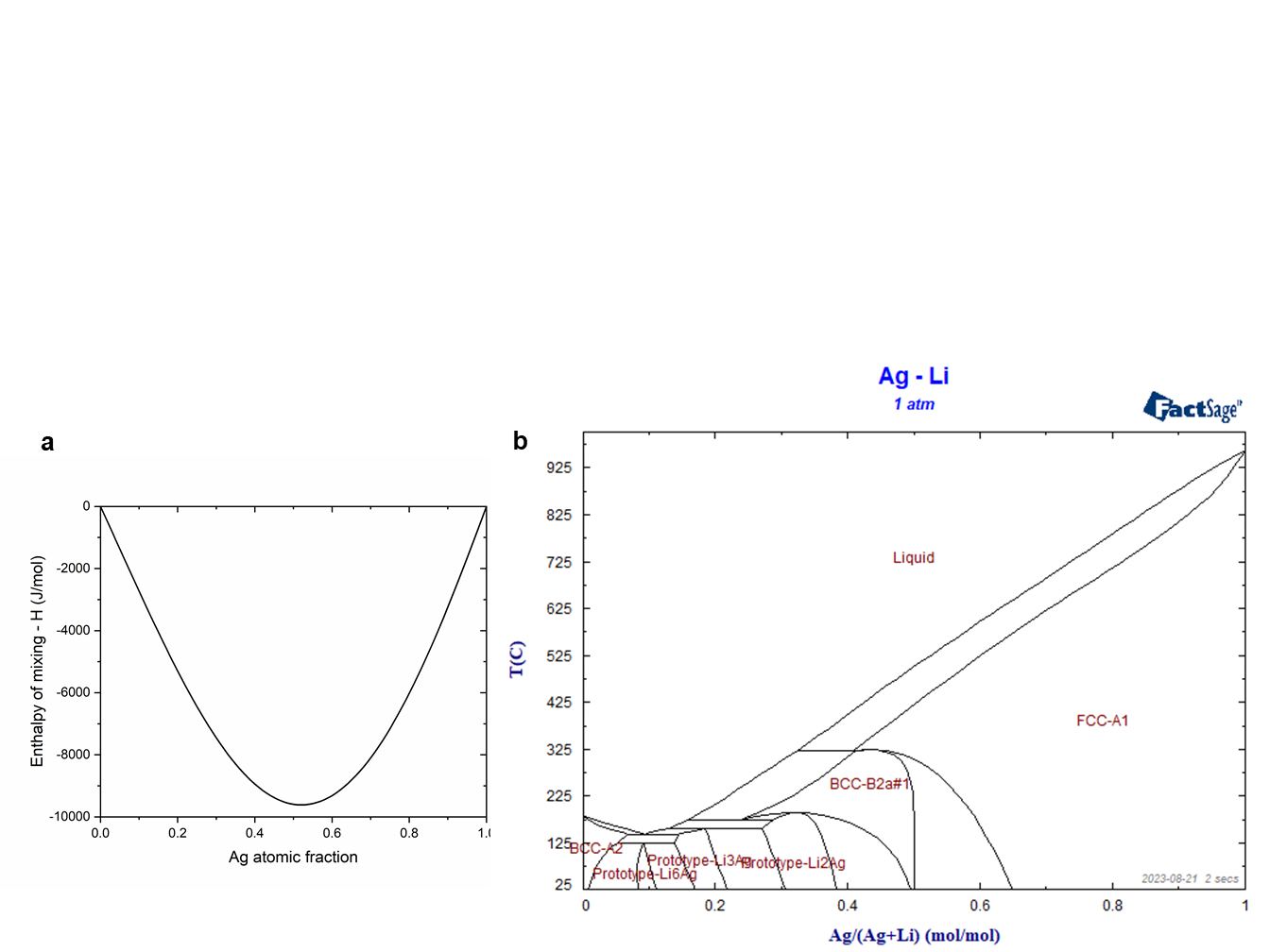


**Supplementary Figure S7 –** (a) Enthalpy of mixing for the FCC-Ag phase as function of the Ag content in the Ag-Li system. The negative enthalpy of mixing throughout the whole range indicates the tendency to form a homogeneous solid solution in the whole composition range. (b) Calculated phase diagram for the Ag-Li system in the FactSage software with the FTLite database.


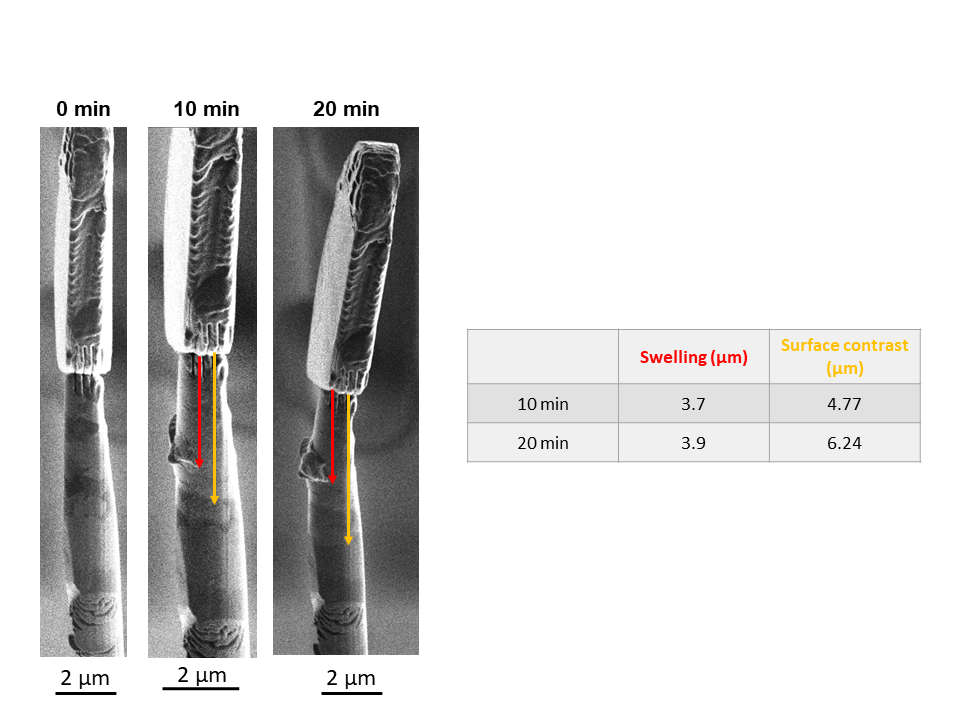


**Supplementary Figure S8 –** Estimation of the diffusion distance for swelling (red arrow) and surface contrast change (orange arrow).


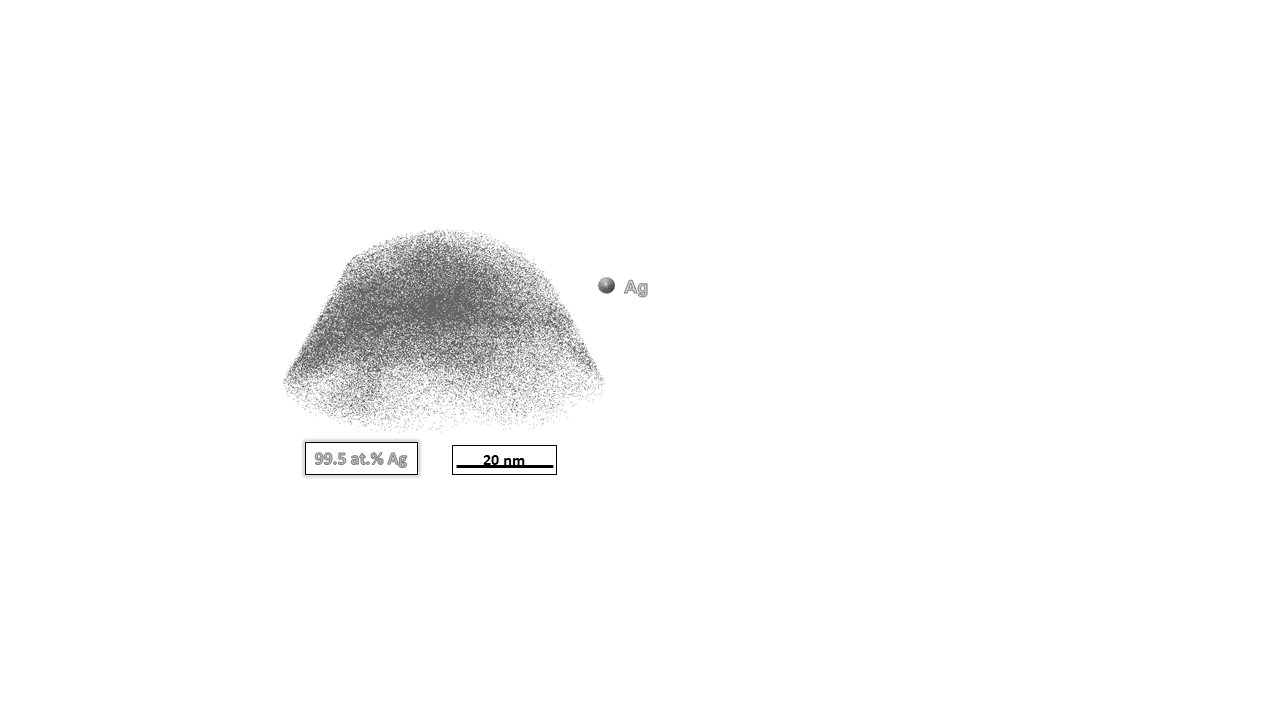


**Supplementary Figure S9 –** APT from (a) pure Ag in the <100> grain after 4 h of reaction in a micro diffusion couple tip. (b) A pure Li region at the top of another <100> grain after 4 h reaction. Only Li plating takes place at the surface of the <100> grain, with the bulk Ag remaining unreacted.


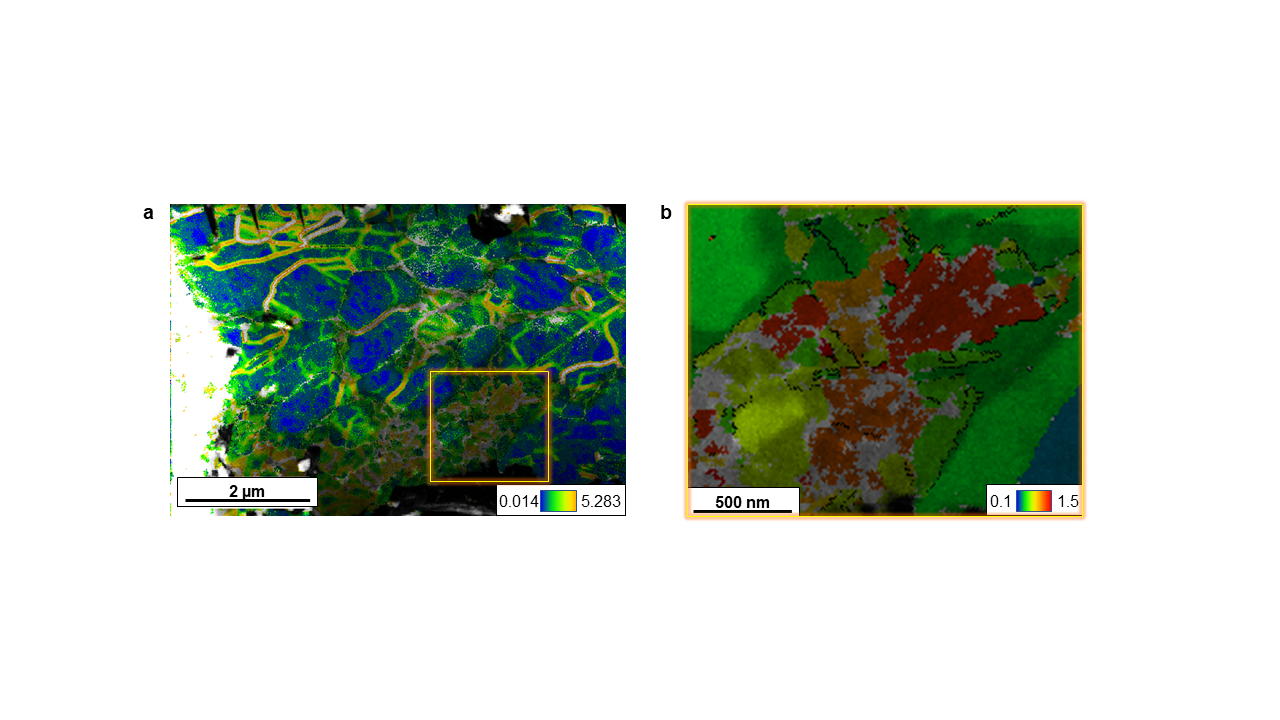


**Supplementary Figure S10 –** (a) Kernel average misorientation (KAM) and (b) grain average misorientation in color scale from the region shown by the orange box in (a), superimposed to Ag EDS signal in gray scale, obtained from the transmission Kikuchi diffraction (TKD) map. In both cases, twin boundaries are shown as black lines. In (b) note the higher KAM in the lithiated regions, indicating the presence of a higher geometrically necessary dislocation density. In (b), twinned lithiated grains exhibit lower grain average misorientation than the surrounding lithiated grains, suggesting the occurrence of dynamic recrystallization mediated by twinning.

**
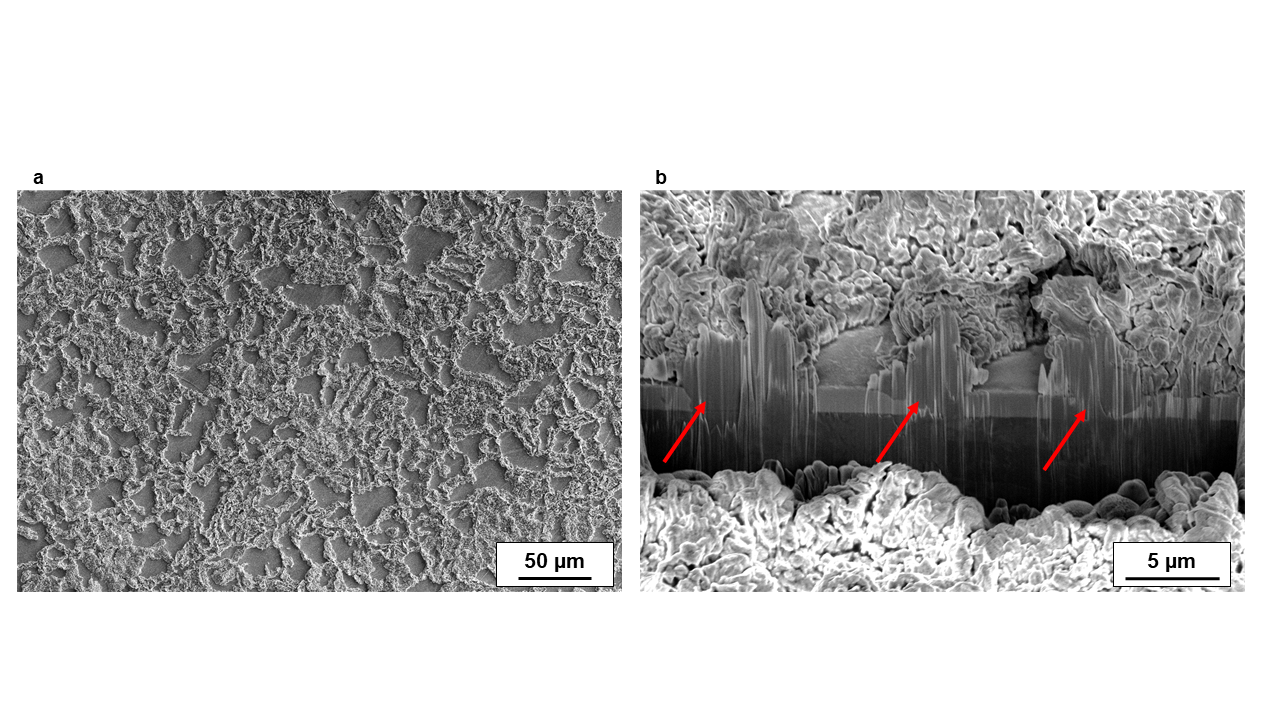
**

**Supplementary Figure S11 –** (a) Top view and (b) cross section of the Ag thin film sputtered onto a 316 stainless steel substrate after lithiation at 50 μA/cm^2^ until a capacity of 0.58 mAh/cm^2^. Notice the lithiated regions indicated by red arrows protruding from the surface in (b), localized at former Ag grain boundaries.


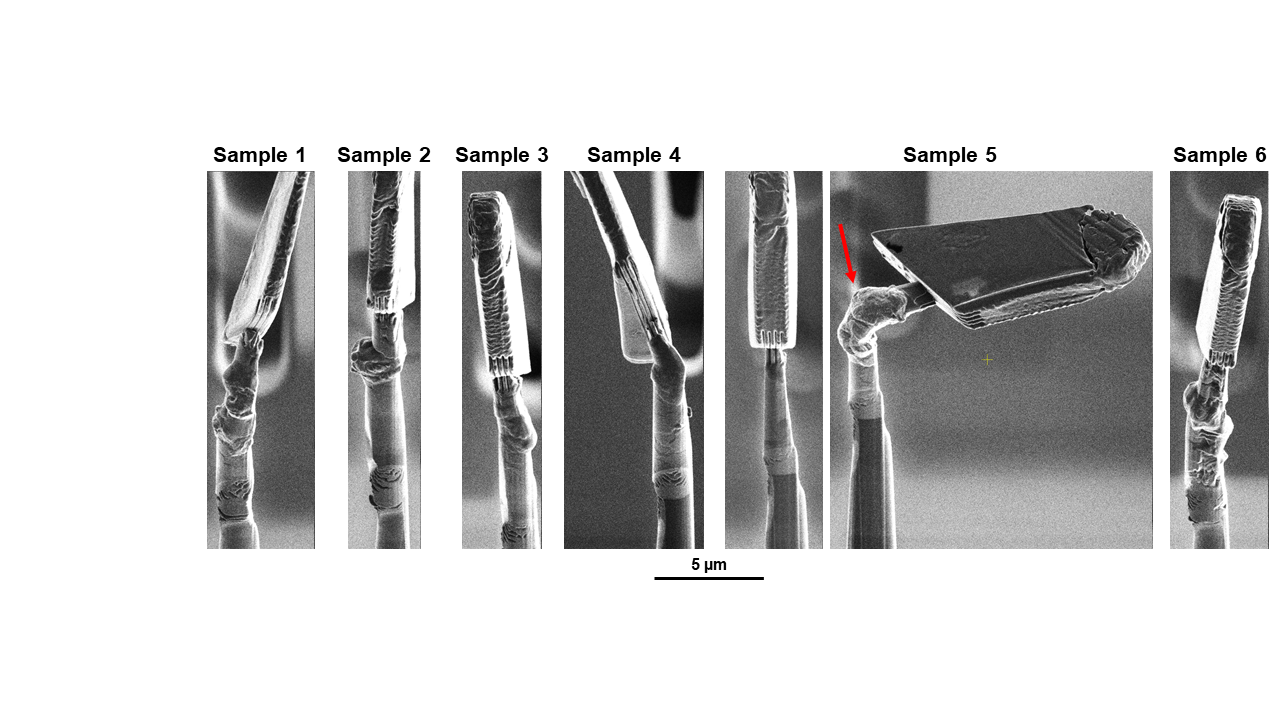


**Supplementary Figure S12 –** Final diffusion couple pillars after reaction and the pronounced localized swelling in sample 5 (408% volume expansion) indicated by the red arrow. Please note the bending of this single pillar that can be attributed to lower mechanical constraint in a pillar with a smaller diameter of 0.9 μm.

**Supplementary Table S1 –** Local volume expansion quantification in grain boundary regions upon lithiation of fine-grained Ag pillars.

| Sample | Initial diameter (µm) | Final diameter (µm) | Height (µm) | Initial volume (µm^3^) | Final volume (µm^3^) | ΔV (%) |
| --- | --- | --- | --- | --- | --- | --- |
| 1 | 1.24 | 1.67 | 1.18 | 1.43 | 2.58 | 81.4 |
| 2 | 1.47 | 2.43 | 1.82 | 3.09 | 8.44 | 173.3 |
| 3 | 1.53 | 1.82 | 1.13 | 2.08 | 2.94 | 41.5 |
| 4 | 1.16 | 1.56 | 1.44 | 1.52 | 2.75 | 80.9 |
| 5 | 0.9 | 2.03 | 2.62 | 1.67 | 8.48 | 408.8 |
| 6 | 1.5 | 2.02 | 1.62 | 2.86 | 5.19 | 81.4 |
